# Thyroidectomy for Cancer: The Surgeon and the Parathyroid Glands Sparing

**DOI:** 10.3390/jcm10194323

**Published:** 2021-09-23

**Authors:** Giuliano Perigli, Fabio Cianchi, Francesco Giudici, Edda Russo, Giulia Fiorenza, Luisa Petrone, Clotilde Sparano, Fabio Staderini, Benedetta Badii, Alessio Morandi

**Affiliations:** 1 Department of Experimental and Clinical Medicine, University of Florence, Largo Brambilla, 6, 50135 Florence, Italy; giuliano.perigli@unifi.it (G.P.); fabio.cianchi@unifi.it (F.C.); edda.russo@unifi.it (E.R.); giulia.fiorenza@yahoo.it (G.F.); fabio.staderini@unifi.it (F.S.); benedettabadii@yahoo.it (B.B.); morandialessio9@gmail.com (A.M.); 2 Department of Biomedical, Experimental and Clinical Sciences Mario Serio, University of Florence, Largo Brambilla, 6, 50135 Florence, Italy; luisa.petrone@unifi.it (L.P.); clotilde.sparano@unifi.it (C.S.)

**Keywords:** thyroid carcinoma, postoperative complications, hypoparathyrodism, hypocalcemia, parathyroid glands, methylene bleu, optical devices, autofluorescence, indocyanine green

## Abstract

Background: The diagnosis of thyroid cancer is continuously increasing and consequently the amount of thyroidectomy. Notwithstanding the actual surgical skill, postoperative hypoparathyroidism still represents its most frequent complication. The aims of the present study are to analyze the rate of postoperative hypoparathyroidism after thyroidectomy, performed for cancer by a single first operator, without any technological aid, and to compare the data to those obtained adopting the most recent technological adjuncts developed to reduce the postoperative hypoparathyroidism. Methods: During the period 1997–2020 at the Endocrine Surgery Unit of the Department of Clinical and Experimental Medicine of the University of Florence, 1648 consecutive extracapsular thyroidectomies for cancer (401 with central compartment node dissection) were performed. The percentage of hypoparathyroidism, temporary or permanent, was recorded both in the first period (Group A) and in the second, most recent period (Group B). Total thyroidectomies were compared either with those with central compartment dissection and lobectomies. Minimally invasive procedures (MIT, MIVAT, some transoral) were also compared with conventional. Fisher’s exact and Chi-square tests were used for comparison of categorical variables. *p* < 0.01 was considered statistically significant. Furthermore, a literature research from PubMed^®^ has been performed, considering the most available tools to better identify parathyroid glands during thyroidectomy, in order to reduce the postoperative hypoparathyroidism. We grouped and analyzed them by technological affinity. Results: On the 1648 thyroidectomies enrolled for the study, the histotype was differentiated in 93.93 % of cases, medullary in 4% and poorly differentiated in the remaining 2.06%. Total extracapsular thyroidectomy and lobectomy were performed respectively in 95.45% and 4.55%. We recorded a total of 318 (19.29%) cases of hypocalcemia, with permanent hypoparathyroidism in 11 (0.66%). In regard to the literature, four categories of tools to facilitate the identification of the parathyroids were identified: (a) vital dye; (b) optical devices; (c) autofluorescence of parathyroids; and (d) autofluorescence enhanced by contrast media. Postoperative hypoparathyroidism had a variable range in the different groups. Conclusions: Our data confirm that the incidence of post-surgical hypoparathyroidism is extremely low in the high volume centers. Its potential reduction adopting technological adjuncts is difficult to estimate, and their cost, together with complexity of application, do not allow immediate routine use. The trend towards increasingly unilateral surgery in thyroid carcinoma, as confirmed by our results in case of lobectomy, is expected to really contribute to a further reduction of postsurgical hypoparathyroidism.

## 1. Introduction

The incidence of thyroid carcinoma has more than tripled in recent decades and consequently so have thyroidectomies and their related complications [1,2]. It has long been known that the most frequent of these is postoperative hypocalcaemia from temporary or permanent parathyroid insufficiency due to unavoidable, involuntary removal, thermal or vascular damage of the parathyroids. The incidence increases if total thyroidectomy is associated with central compartment lymph-node dissection, when parathyroid glands are often accidentally or inevitably removed in the pursuit of oncological radicality [3,4]. The temporary form is still frequent and the permanent form is problematic to treat due to the potential negative aspects of prolonged administration of calcium and vitamin D and the unavailability of a replacement hormone [5,6]. Considering earlier diagnosis with smaller size of thyroid carcinoma, the most recent guidelines point towards less aggressive and often unilateral surgical treatment [1,2,3,4,5,6,7]. The expected reduction in hypoparathyroidism is not substantial. In fact, total thyroidectomy remains by far the most prevalent intervention due to the presence of concomitant contralateral nodularity or hormonal hyperfunction [8]. Moreover, even in lesions with indication for radiometabolic therapeutic completion, surgical radicality should always be pursued to avoid potential interferences in the humoral and instrumental follow-up of a parenchymal residual. Although hypoparathyroidism is the most common complication after thyroidectomy, the literature reports extremely different incidence and prevalence values, ranging from 1.6% to more than 50%. In literature, the adoption of non-univocal and non-standardized parameters in reporting postoperative complications determines the inclusion in the different case series of very heterogeneous patients, symptomatic or asymptomatic, with mild or severe hypocalcaemia [9,10,11]. Especially in the case of permanent hypoparathyroidism, which worsens the quality of life due to replacement therapy and undefined controls, the differences are even more marked if one compares the case histories of dedicated surgical centres, general centres and general epidemiological surveys including patients who have escaped specialist controls. In fact, contrary to what has been estimated, the majority of the studies report hypoparathyroidism with percentages of more than 10%, even though these are often mild forms that can be easily controlled with low doses of calcium and vitamin D, which rarely expose the patient to complications such as calcification of the extra-skeletal soft tissues, basal ganglia and kidney, as observed in cases that require much higher doses to compensate for the almost total lack of parathyroid hormone [12,13,14,15,16,17,18].

In the face of these unexpected rates of post-surgical hypoparathyroidism revealed by the most recent studies, it is not surprising that surgeons have turned to testing every means of reducing them. In fact, it no longer seemed sufficient to rely solely on the recommendations of good surgical practice and the individual surgeon’s experience and ability to detect them with the naked eye aided only by good lighting and optional optical magnification as basically indicated by the most authoritative guidelines [1].

The present paper, with a mainly clinical focus, has two aims: (a) to analyze the rate of postoperative hypoparathyroidism after thyroidectomy performed for cancer by a single first operator without any technological aid; (b) to evaluate if the numerous technological proposals that have emerged in recent years in an attempt to make objective identification of the parathyroids and assessment of their function, overcoming the limits related to the subjective judgement of the individual surgeon, are really useful in reducing the postoperative hypoparathyroidism incidence.

## 2. Materials and Methods

During the period 1997–2020 at the Endocrine Surgery Unit of the Department of Clinical and Experimental Medicine of the University of Florence, 1648 consecutive extracapsular thyroidectomies for cancer (401 with central compartment node dissection) were performed, and the patients’ data prospectively recorded in an electronic database. A prospective study was conducted after approval by the Area Vasta Regione Toscana/AOUC Ethics Committee (N 20534). An informed written consent was obtained from each patient.

For the follow-up we have collaborated with the Endocrinology Unit of the same institution with which this activity has been constantly shared.

These are 1648 consecutive thyroidectomies, in 401 patients with central compartment lymph node dissection, to treat thyroid carcinoma performed almost exclusively by one of the authors (GP) in little more than 20 years and in small part (<10%) by collaborators always in his presence.

The case series was divided into a first period (March 1997–April 2015) (Group A) and a second period (May 2015–December 2020) (Group B), each consisting of 824 consecutive cases. No technological adjunct was adopted in both periods. The thyroidectomy procedure, always extracapsular, was carried out with the naked eye without magnifying glasses or frontal light but only with the operating light.

In addition to demographic data, the percentages of hypoparathyroidism (symptomatic or asymptomatic), temporary or definitive, were recorded calcium and PTH values, tested preoperatively, and at 12, 18 h and 7 days postoperatively lower than normal (respectively 8.5 mg/dL and 1.5 pmol/L in our laboratory) and the need for calcium-vitamin D replacement therapy over six months after surgery for the definitive form.

We also included the 75 lobectomies in which the central compartment had been explored and compared with total extracapsular thyroidectomies.

Total thyroidectomies were compared with those with central compartment and laterocervical lymph-adenectomy (in which we systematically also performed the central dissection); minimally invasive procedures (MIT, MIVAT, some transoral) with conventional ones and the first period of the series with the second one.

For the statistical analysis, Chi-square tests or Fisher’s exact, when appropriate, and were used for comparison of categorical variables. *p* < 0.01 was considered statistically significant.

From the literature published in Pub Med^®^ in recent years, we extracted the most suitable experiments to represent the current means available for a better identification of the parathyroid glands during thyroidectomy. We grouped them by technological affinity. The most reliable in terms of potential immediate clinical use experiments were critically revised.

## 3. Results

### 3.1. Our Experience

On 5264 thyroidectomies performed from January 1997 to December 2020, we enrolled all the 1648 patients who had undergone thyroidectomy for carcinoma. The histotype was differentiated in 93.93% of cases, medullary in 4% and poorly differentiated in the remaining 2.06%.

In 95.45% of the cases total extracapsular glandular excisions were performed and in 4.55% lobectomy alone was considered oncologically sufficient after negative exploration of the central compartment.

We recorded a total of 318 hypocalcemia (19.29%) of which 11 (0.66%) diagnosed as permanent hypoparathyroidism requiring therapy for more than 6 months. In particular, we found that hypocalcemia affected 316 patients after total thyroidectomy (20.09%), and only 2 patients after lobectomy (2.66%); after total thyroidectomy, 202 patients (25.06%) had hypocalcemia in Period A and 114 (14.86%) in Period B (*p* < 0.0001), while no patient in Period A and 2 patients (3.51%) in Period B suffered this complication after lobectomy (*p* < 0.0001).

**Figure 1:**
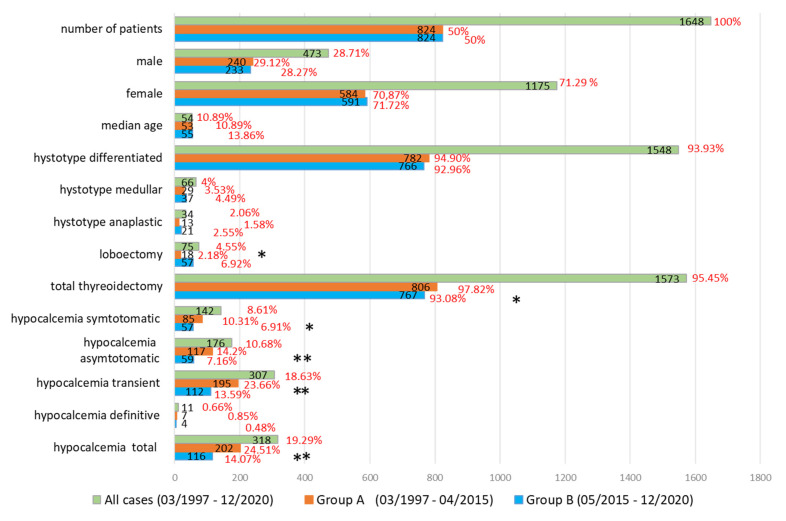
Demographic characteristics of the patients, type of operation and histotype of malignancy, incidences of temporary and permanent hypocalcaemia in the total case series and in the first and second period. Black numbers indicate the number of patients, while red numbers indicate the percentage. Differences between the groups were calculated via the Chi Square, or Fisher’s exact test, when appropriate (both 2-tailed); a *p* < 0.01 is considered statistically significant. * *p* < 0.01, ** *p*< 0.0001.

Clinical characteristics of patients are described in Figure 1 and Table 1.

### 3.2. Literature Examination

The literature reports four categories of tools that can be used by the surgeon, together with his eyes and experience, to facilitate the identification of the parathyroids (Table 2):

(a) Vital dye such as methylene bleu [19,20,21].
(b) Optical devices without contrast media and unaffected by ambient light [22,23,24,25,26].
(c) Autofluorescence of parathyroids detected by infrared light or laser stimulation [27,28,29,30,31,32,33].
(d) Autofluorescence enhanced by injection of indocyanine green or 5-ALA [34,35].

## 4. Discussion

The literature evaluation has enabled us to consolidate certain convictions developed over many years of activity. Firstly, the difficulty of defining hypoparathyroidism due to the variables that characterize it and the clinical manifestations that arise with serum calcium levels, which vary greatly in every single patient [1].

We agree with Sitges-Serra that it is inappropriate and reductive to categorize it clearly between clinical and humoral, temporary and definitive, since functional recovery is a dynamic process that can last up to two years [9].

We have noted a considerable discrepancy in the prevalence of post-surgical hypoparathyroidism, especially permanent, reported in the various case histories according to their origin. The ranges appear almost irreconcilable when comparing the surgical series with the endocrinological or epidemiological ones, and the most common values are around 1–3% in the former and around 12% in the latter [3,8,10,15,36].

There is a unanimous agreement in the literature that the identification and functional preservation of the parathyroids is difficult due to the presence of contiguous similar structures such as thyroid nodules, lymph nodes, adipose lobules or fibrosis from previous operations. Therefore, it is directly related to the sensitivity and experience of the surgeon, even though he strictly adheres to the principles of good surgical practice, which, in addition to anatomical integrity and the number of glands identified, also recommend respect for vascular support [1,37,38]. Recent studies have in fact shown that accidental removal and tissue or vascular damage do not find a remedy in glands autotransplantation [9], and allografting is still not beyond the experimental stage or good hopes for the future [39]. There is evidence that systematic and meticulous research can lead to invisible parathyroids damage and that selective identification is preferable to routine identification. In fact, it would appear that the number of parathyroids actually left ‘in situ’ is more important than the number of those identified [8,40,41,42,43].

Regarding instruments potentially useful in reducing post-surgical hypocalcaemia through better identification and functional preservation of the parathyroids, intraoperative biopsy, rapid PTH dosage on aspirate [44,45,46,47] and gamma probe identification [48], which were proposed in the past and are now obsolete because they are invasive, costly, time-consuming and ethically inapplicable as they damage tissue that should ideally be preserved as much as possible, are now rarely used.

The simplest and easiest means of identifying vital parathyroids would be the use of methylene blue. Known for fifty years [19] and appreciated for the identification of pathological glands, it did not provide the same results in the recognition of normal glands during thyroidectomy when injected intravenously. The recent adoption of a spray application directly on the operating field seems to have achieved a high level of accuracy, even avoiding the problem of the potential toxicity [20,21,49].

Looking at the group of instruments based on optical technology such as DOCI (dynamic optical contrast imaging), FLIm (fluorescence lifetime imaging), LSCI (laser speckle contrast imaging), NIMI (near infrared molecular imaging), LIBS (laser induced breakdown spectroscopy), it is immediately evident that, despite the advantage of not using any contrast medium and not being influenced by ambient light, these are still experimental applications and reserved for research centres with strong financial backing, certainly not within the reach of most thyroid surgery centres [22,23,24,25,26,33]. They demonstrate a high level of accuracy in identifying and assessing glandular perfusion, but cannot prevent any iatrogenic damage produced during retrieval, which in any case precedes the test. Due to their complexity, they appear to be far from an imminent clinical application.

Paras et al. were the first in 2011 to discover and describe [27] an autofluorescence of the parathyroids induced by stimulation with high-energy light sources of endogenous fluorophores that reacted by emitting low-energy light. The exploitation of this property, apparently easier to apply and with potentially more immediate advantages, has offered a new opportunity in the attempt to reduce post-surgical hypoparathyroidism [27,28,29,30,31,32,33].

The long persistence of autofluorescence in parathyroids even after their removal makes this property unsuitable for perfusion assessment and has necessitated the injection of exogenous fluorophores as contrast agents or dyes (ICG, indocyanine green; 5-ALA, 5-aminolevulinic acid) to enhance natural fluorescence.

There are other commercially available laparoscopic or handheld camera instruments which, although designed for ICG study of other organ perfusion or sentinel node detection, can be used in parathyroid fluorescence detection. Even instruments combining two methods (Niraf and Laser-speckle contrast Imaging), Ref. [33] are not yet fully convincing and in any case not applicable on a large scale.

Solorzano et al., examined in detail all fluorescence-based technology applicable to parathyroid surgery today and compiled a valuable list of possible indications with potential advantages and disadvantages specific to probe-based and chamber-based technology.

Although the literature reports a sensitivity of autofluorescence of 80 to 100%, it is also correctly acknowledged that there are false positives and negatives and that the main limitation of the method lies in its poor tissues penetration power and that it requires the manipulation of parathyroids, exposing to tissue damage. The additional use of ICG improves the power of identification and allows a judgement of perfusion that is not possible with autofluorescence alone. The conclusions are not definitive, so we invite others to further clarify its real cost–benefit [50].

To date, unfortunately only a few studies have demonstrated a direct correlation between visualization and glandular perfusion and a reduction in hypoparathyroidism [30], which is in any case limited to the temporary but not definitive form. Moreover, none of the prevention methods adopted seem to be able to reduce hypocalcaemia [42,49,50,51,52,53,54,55].

In summary, alongside with authoritative reviews confirming the feasibility and efficacy of recent parathyroid identification aids [56,57,58], there are others that urge caution in adopting them too enthusiastically before larger prospective and randomised studies confirm their superiority over surgeon volume and skill [59,60]. For example, the analysis of our case history, while showing values for temporary hypoparathyroidism that are in line with the literature (19.29%), confirms the negligible values of our previous investigations [4,8,18,61,62], for permanent hypoparathyroidism. The strict adoption of the specific principles of thyroid surgery required by the university didactic nature of our hospital and the high volume of cases treated, we believe, is sufficient to justify values much lower than those of other series but not too dissimilar from other Italian multicentric experiences where values of temporary and permanent hypoparathyroidism of 8.3% and 1.7% respectively are reported [3].

In this regard, the comparison between the first and second part of our series seems very expressive: although they are absolutely superimposable in terms of demographic characteristics and the methods adopted, they reveal statistically significant differences for both forms of hypoparathyroidism, confirming the well-known and recently reaffirmed [14] relationship between the surgeon’s case-volume and the number of complications.

As expected, and as already noted in our previous experiences, the differences between minimally invasive and conventional surgery in the two groups are not significant [61,62].

In the second group, in accordance with the most recent guidelines, lobectomies increased but hypocalcaemia, which was never definitive, remained negligible, confirming that unilateral surgery, even in the cases we included with exploration of the central compartment, protects against hypocalcaemic complications.

On the other hand, the values are very different in both groups when comparing simple thyroidectomies and those accompanied by lymphectomy of the central compartment, respectively 19.41% vs. 38.8% in Group A and 8.61% vs. 32.97% in Group B. Interestingly, the percentages of hypoparathyroidism remained very similar in the two periods when total thyroidectomy was accompanied by central compartment lymphectomy, 38.88% in Group A and 32.97% in Group B, respectively. Moreover, out of eleven patients with definitive hypoparathyroidism, as many as eight had undergone central compartment lymphectomy, demonstrating that the complication is strictly procedure-dependent and cannot be modified by the surgeon’s experience.

In addition, this study also confirms the higher incidence of hypocalcaemia in simple total thyroidectomies for carcinoma compared with total thyroidectomies for benign disease, as previously reported by us and other authors (19.29% vs. 12.99), [4,8,14,63]. However, a recent article from Onder CE et al., 2020, reports that the management of the patients with hypocalcaemia is suboptimal with active vitamin D and cholecalciferol treatment [64].

The limitation common to all the examined tools is that they only record what has already taken place and are not able to modify the intra-operative procedure except to indicate an autotransplant in the event of hypoperfusion, with the possibility to increase the risk of hypocalcaemia [9], therefore, the surgeon remains the one who has to assess the parathyroids site, shape, color (and its possible variations) or, by touch, their consistency.

## 5. Conclusions

Our findings show that post-surgical hypoparathyroidism is extremely uncommon in high-volume institutions. Its potential decrease through the employment of technical adjuncts is impossible to quantify, and their expense, combined with the complexity of their application, makes them unsuitable for immediate usage. As evidenced by our findings in the case of lobectomy, the trend toward more unilateral thyroid surgery is predicted to lead to a further reduction in postsurgical hypoparathyroidism.

## Figures and Tables

**Table 1:** Comparison of conventional and minimally invasive procedures and of patients who received only a total thyroidectomy versus those in whom a central compartment lymphectomy was also performed, whether or not extended to the lateral compartments.

		**Group A**		**Group B**		***p* Value**	**Group A**		**Group B**		***p* Value**
**Type of Surgery**		**Minimally Invasive**					**Conventional**				
cases		403		257			421		567		
		*N.*	*%*	*N.*	*%*		*N.*	*%*	*N.*	*%*	
hypocalcemia	symptomatic	38	9.43	14	5.44	0.0752	47	11.16	43	7.58	0.0577
	asymptomatic	56	13.89	24	9.34	0.0875	61	14.49	35	6.17	<0.0001
	transient	91	22.58	37	14.39	0.01	104	24.7	76	13.4	<0.0001
	definitive	3	0.74	1	0.39	1	4	0.95	2	0.35	0.4106
	total	94	23.32	38	14.78	0.003	108	25.65	78	13.75	<0.0001
**Type of Surgery**		**Cclnd**					**No Cclnd**				
cases		216		185			608		639		
		*N.*	*%*	*N.*	*%*		*N.*	*%*	*N.*	*%*	
hypocalcemia	symptomatic	44	20.37	32	17.29	0.4464	45	7.4	28	4.38	0.0293
	asymptomatic	40	18.51	29	15.68	0.5077	73	12.01	27	4.22	<0.0001
	transient	79	36.57	59	31.89	0.3	116	19.08	53	8.29	<0.0001
	definitive	5	2.31	2	1.08	0.4589	2	0.33	2	0.31	1
	total	84	38.88	61	32.97	0.2	118	19.41	55	8.6	<0.0001

Abbreviations: cclnd, central compartment lymph node dissection. Differences between the groups were calculated via the Chi Square, or Fisher’s exact test, when appropriate (both two-tailed); a *p* < 0.01 is considered statistically significant.

**Table 2:** The main original studies evaluating technologies in order to avoid postoperative hypoparathyroidism, from 1971 to 2021.

Reference	Technology	Article Type	Nb pt	Parathyroid Identification	Postoperative Hypo-PTH /Hypoca	Conclusions
Dudley et al. [19] 1971	Intravenous infusion of methylene blue	original/humans	17	41/68	/	Could help to reduce the high incidence of clinical hypoparathyroidism after total thyroidectomy.
Monib et al. [20] 2020	Intraoperative methylene blue spray	original/humans	50	82% accuracy	18%	Safe, feasible, and effective to identify parathyroid glands
Sari et al. [21] 2012	Intraoperative methylene blue spray	original/humans	56	/	5% transient	Identification of parathyroid glands within three minutes and also of recurrent laryngeal nerves and inferior thyroid arteries.
Hu et al. [22] 2021	Dynamic optical contrast imaging (DOCI)	original/animals and humans ex vivo	/	/	/	Facilitates specific parathyroid gland localization
Marsden et al. [23] 2021	Fluorescence lifetime imaging (FLIm)	original/humans	21	100% sensitivity 93% specificity	/	Good sensitivity and specificity for the rapid identification of PG.
Mannoh et al. [24] 2021	Laser speckle contrast imaging (LSCI)	original/humans	72	/	8.3% temporary 1.4% permanent	Promising technique for assessing parathyroid gland vascularity
Kennedy et al. 2021 [25]	Near-infrared molecular Imaging (IMI)	original/humans	5	9/9	1/9 asymptomatic	Accurate and reproducible method of localizing parathyroid glands
Wang et al. [26] 2021	Laser-induced breakdown spectroscopy (LIBS)	original/animals ex vivo	/	/	/	Can discriminate between smear samples of PG and NPG
Paras et al. [27] 2011	Near-infrared (NIR) autofluorescence	original/humans	21	/	/	Parathyroid fluorescence was two to eleven times higher than that of the thyroid tissues with peak fluorescence occurring at 820 to 830 nm.
Aoyama et al. [28] 2020	Near-infrared (NIR) autofluorescence	original/humans	2	/	/	The autofluorescence of diseased glands was weaker than that of normal glands, even with the excitation light of NIR.
Akbulut et al. [29] 2021	Near-infrared (NIR) autofluorescence	original/humans	300	25% *	/	Second-generation NIFI (CMOS) displayed higher detection rates and AF intensity.
Kim et al. [30] 2021	Near-infrared (NIR) autofluorescence	original/humans	542	/	4.2% permanent	May reduce temporary hypoparathyroidism and the risk of inadvertent resection of PGs in CND.
Wiseman et al. [31] 2021	Near-infrared (NIR) autofluorescence	original/humans in vivo and ex vivo	/	/	/	Can successfully intraoperatively identify both normal and pathological PGs.
Kiernan et al. [32] 2021	Near-infrared (NIR) autofluorescence	original/humans	83	94.3% accuracy	/	Probe-based NIRAF detection can be a valuable adjunct device to intraoperatively identify PGs.
Mannoh et al. [33] 2021	ParaSPAI a device that combines NIRAF imaging with LSCI	original/humans	/	/	/	Capable of label-free parathyroid gland identification and vascularity assessment through the combination of NIRAF imaging with LSCI.
Suzuki et al. [34] 2011	5-Aminolevulinic Acid	original/humans	13	In all patients at least one	/	Useful to localize the normal parathyroid glands during thyroid surgery
Jin et al. [35] 2018	Indocyanine green	original/humans	26	/	7.69% transient	Safe, easy and effective method to protect the parathyroid and predict postoperative hypoparathyroidism

Abbreviations: / = no available data. Nb pt = number of patients; PG = parathyroid gland; NPG = no parathyroid gland; * = before visual identification of PGs; NIFI = near-infrared fluorescence imaging; CMOS = complementary metal-oxide semiconductor; AF = autofluorescence; CND = central neck dissection; ParaSPAI = parathyroid speckle and autofluorescence imager; LSCI = laser speckle contrast imaging.
